# Implementation of a Practice Development Model to Reduce the Wait for Autism Spectrum Diagnosis in Adults

**DOI:** 10.1007/s10803-018-3501-5

**Published:** 2018-03-03

**Authors:** Marion Rutherford, Kirsty Forsyth, Karen McKenzie, Iain McClure, Aja Murray, Deborah McCartney, Linda Irvine, Anne O’Hare

**Affiliations:** 1 School of Health Sciences, Queen Margaret University, Queen Margaret University Drive, Edinburgh, EH21 6UU Scotland, UK; 2 NHS Lothian Speech and Language Therapy Department, Royal Hospital for Sick Children, 5 Rillbank Terrace, Edinburgh, EH9 1LS Scotland, UK; 3 Faculty of Health and Life Sciences, Northumbria University, Newcastle Upon Tyne, UK; 4 School of Clinical Sciences, University of Edinburgh, Edinburgh, Scotland, UK; 5 Institute of Criminology, University of Cambridge, Cambridge, UK; 6 NHS Lothian, Child and Adolescent Psychiatry, Edenhall Hospital, Musselburgh, UK; 7 NHS Lothian Headquarters, Waverley Gait, Edinburgh, Scotland, UK

**Keywords:** ASD, Adults, Diagnostic assessment, Reducing wait times, Service improvement

## Abstract

This study examined waiting times for diagnostic assessment of Autism Spectrum Disorder in 11 adult services, prior to and following the implementation of a 12 month change program. Methods to support change are reported and a multi-level modelling approach determined the effect of the change program on overall wait times. Results were statistically significant (b = − 0.25, t(136) = − 2.88, p = 0.005). The average time individuals waited for diagnosis across all services reduced from 149.4 days prior to the change program and 119.5 days after it, with an average reduction of 29.9 days overall. This innovative intervention provides a promising framework for service improvement to reduce the wait for diagnostic assessment of ASD in adults across the range of spectrum presentations.

## Introduction

### Delay in ASD Diagnosis in Adults

Late and delayed identification of those in the adult population with an Autism Spectrum Disorder (ASD) poses an international challenge (WHO 2013), with the mean age of diagnosis in adults reported to be between 31 and 49 years (e.g. Rutherford et al. 2016a; Wilson et al. 2013). A growing number of adults are being referred for assessment and diagnosis of ASD (Happé et al. 2016) and for adults with ASD who experience difficulty in their day to day life, waiting for a diagnosis means going without support and/or interventions that can improve overall health and wellbeing (Powell 2002; Scottish Government 2011; WHO 2013).

ASD diagnostic assessment for adults is largely provided by mental health services, where it is known that success of treatment depends on timely access (Goin and Myers 2004; Kowalewski et al. 2011). Delayed diagnosis is an indicator of the need for improvement to the health care system (Sherman et al. 2009) and lengthy diagnostic processes have a knock on effect on both society and the people waiting for a diagnosis (Gallucci et al. 2014; Osborne and Reed 2008). It is reported that long waits threaten public confidence in medical care and have been shown to increase morbidity and mortality of mental health service users (Haggarty and Jarva 2012, p. 30). The majority of adults with ASD have poor outcomes (Henninger and Taylor 2013) and delayed diagnosis impacts on this.

Although the number of adults on ASD diagnostic service waiting lists is rising (Jantz 2011), adding to the length of time they wait for diagnostic assessment, there is currently no recommended standard for the maximum wait time for ASD diagnostic assessment in adults. The only comparator is the UK standard for children, which recommends a time scale of 119 days from referral to diagnosis (Le Couteur 2003). In a recent study, the average total wait for an adult ASD diagnosis from referral to diagnosis was 162 days (range 14–511 days) and 59% of adult cases exceeded 119 days (McKenzie et al. 2015). Within mental health services, measurements of delayed access to assessment have focused on the wait between referral and access to mental health services for both children and adults (Haggarty and Jarva 2012; Kowalewski et al. 2011).

In recent years, the cause of delayed access to mental health services has been attributed to both individual and environmental factors; for example: working with limited resources and not enough manpower (Sherman et al. 2009); demand for mental health services outstripping resources (Mireau and Inch 2009); limited providers with specialised training (Duffy et al. 2002) and missed appointments (Sherman et al. 2009). Although several studies have explored wait times in child services (Rutherford et al. 2017), minimal research has focused on adults. Previous research in Scotland, which investigated the reasons for diagnostic delay in adults with ASD (McKenzie et al. 2015) identified factors associated with longer assessment duration, for example: the presence of a risk factor for ASD, such as having an intellectual disability, or a neurological disorder; or a greater number of contacts with clinicians. The authors suggest that this may be due to increased clinical complexity in these cases, and propose that this complexity may be reduced by using standardised assessments, clearer pathways, and proformas to gather diagnostic information (McKenzie et al. 2015; Rutherford et al. 2016b).

### How to Reduce Delays

Service change, such as the work reported here, to reduce delays is more effective when informed by theory (Melton et al. 2012; Steinmo et al. 2015). Two key elements in reducing delays are identified in the literature:

### Identify Targets for Intervention

Firstly, delays can be reduced if specific issues are identified and targeted for change (Dobbins et al. 2009). Such changes should be focused on the adoption of evidence based practice (Drake et al. 2001). The National Institute of Clinical Excellence (NICE) (2012) has developed guidelines on the identification, diagnosis and management of ASD in adults which aims to improve care for this client group and identifies the challenge of delayed diagnosis. The required changes targeted within the present study were identified through review of evidence from services, including local actions plans (Rutherford et al. 2016b) and consideration of adherence to these NICE guidelines (McKenzie et al. 2016). A more detailed description of the intervention is outlined in the methods section below.

### Design and Implement the Intervention

Secondly, reducing delays requires putting in place a change program to support practitioners to make the required changes, as research suggests that knowledge of what needs to change in practice does not automatically equate to actual change (Melton et al. 2010). To do this effectively requires skill development, the adoption of new routines in practice and the motivation to embrace the opportunities for new ways of working (Holmes and Scaffa 2009). To the authors’ knowledge no research exists to date, on the application of a specific model of service change for services which diagnose ASD, with the aim of reducing waiting time for diagnosis.

### A Framework for Practice Development

A range of implementation frameworks have been applied in healthcare, which outline key principles and systematic steps required by the complex, dynamic process of facilitating improvements (Meyers et al. 2012). One such framework for change, named “Flightgate” (Melton et al. 2010) has been based upon the Individual Practice Development theory (Melton et al. 2012) and has been used previously within mental health service change programs (Melton et al. 2010). This theory promotes self reflection and the selection of, and engagement in, differentiated, targeted activities to support change in practice. The Flightgate practice development program is underpinned by the position that any change process needs to have available (a) practice development mentorship, (b) peer group forums, (c) practise in practice, and (d) public validation roles.

*Practice development mentorship* is fundamental to this conceptual framework. Although diagnostic practitioners possess enhanced clinical expertise and local knowledge, research indicates the need for support in developing a deeper understanding of effective implementation of change—i.e. what should be modified and how (Meyers et al. 2012). Specific mentorship aims to build confidence and support staff in “finding flow” or removing barriers to adopting new practices (Melton et al. 2012).

*Peer group forums*, used as a means of conferring with others, are known catalysts for practice change. Several studies report on the benefits of using active and interpersonal knowledge sharing techniques and the benefit of tailoring these to specific audiences (e.g. in Harrington et al. 2008; National Center for the Dissemination of Disability Research [NCDDR], 2006). The influence of networks on the successful sharing of knowledge is noted and informal electronic networks offering targeted e-mails highlighting new research information or evidence was perceived to be a highly valuable and legitimate knowledge sharing strategy among health professionals (Russell et al. 2004).

*The strategy of “practise in practice”* is based on the premise that practical tools can promote skill development (Melton et al. 2012). Additionally, the successful use of new skills requires the adoption of new routines in practice, together with the motivation to embrace the opportunities for new ways of working (Holmes and Scaffa 2009). To succeed, staff must find effective ways of channelling time (Melton et al. 2012) which is supported through mentorship. In addition to making resources physically accessible, ensuring the clear and concise presentation of research evidence has been identified as vital in improving the probability of its use (Harrington et al. 2008; Mitton et al. 2007; Pyra 2003). This is further illustrated by findings that knowledge sharing methods should be flexible enough to provide users with access to research evidence in various formats and levels of detail to meet individual preferences and need (Dobbins et al. 2004).

Systematic reviews of interventions to promote the implementation of research findings (Bero et al. 1998; Fixsen et al. 2005) identified evidence for the effectiveness of a number of different approaches to supporting change: face-to-face methods including educational outreach visits, reminders of research findings and multifaceted interventions including combinations of audit and feedback.

It has been argued that limiting knowledge sharing methods to the provision of educational materials or didactic educational methods has been shown to have minimal effect, a finding corroborated by a meta-synthesis of systematic reviews into interventions to change health practitioners’ behaviours in response to new knowledge (Grimshaw et al. 2001). Indeed, this overview also identified the effectiveness of multifaceted and active educational approaches. In the change program reported in the current study, one role of the facilitators would be to support practise in practice through the provision or sharing of practical tools and proformas and making research evidence accessible and usable.

Public validation is the fourth strand of the framework and is based on the assertion from the Individual Practice Development theory that accumulating reward over time is important to sustaining change (Forsyth et al. 2005, 2014; Melton et al. 2012). Practitioners commonly feel devalued, without recognition of effort. Validation can take many forms, such as taking on leadership roles, training and supervising others, peer-recognition from colleagues, and positive feedback.

This study is, therefore, seeking to understand if waiting time for ASD diagnosis could be reduced by implementing the Flightgate practice development interventions alongside the Autism Achieve Alliance local action plans (Rutherford et al. 2016b).

## Method

### Governance Processes

The NHS Scotland Caldicott Guardian process provided national approval for the data gathering protocol to gather secondary data. The National Health Service Research and Development Departments of each of the participating services also granted approval for this study.

### Waiting for ASD Diagnosis

For service users and their families, their perception of the ‘wait’ is likely to include the time from referral until they receive a diagnosis. The wait for ASD diagnosis was, therefore, defined to include the period from referral for ASD assessment to being told about their diagnosis. This was inclusive of (a) referral to the first appointment; (b) the duration of ASD diagnostic assessment (from the first appointment to the last appointment) and (c) the ‘wait to receive a diagnosis’, which was the time taken to receive the diagnosis after the last appointment was recorded.

### Services

Participating services were identified from the Diagnosing Services National List (Autism Achieve Alliance AAA 2012) which acted as the sampling frame of all services providing assessment and diagnosis of adults with ASD in Scotland. This list identified 32 adult ASD diagnostic services all of whom were invited to participate in the study. Of these 32, 19% (6/32) were unresponsive and of the 26 remaining, 42% (11/26) chose to participate. Of these 73% (8/11) were Intellectual Disability (ID) Services, 18% (2/11) were Mental Health Services and 9% (1/11) was a specialist ASD service. The 11 adult services (Table 1) averaged 5.5 multi-disciplinary team (MDT) members per service (range 1–11 members). Of the 11 services, 9% (1/11) had a single core ASD diagnosing practitioner, 18% (2/11) had two core practitioners, 9% (1/11) had 3 core practitioners, and 64% (7/11) included 5 or more practitioners. The following professionals were included in the teams: 73% (8/11) had a Clinical Psychologist; 64% (7/11) had a Speech and Language Therapist; 36% (4/11) had an Adult Psychiatrist; 27% (3/11) had a Specialist Nurse; 27% (3/11) had an Occupational Therapist; 9% (1/11) had a Physiotherapist.

**Table 1:** Service configuration of participating adult services

Service	Service type	Urban/rural area	Health board region, and population size	Type of assessment	Number of diagnostic team staff	Professions represented in the wider team undertaking assessment contributing to the diagnosis (lead professional in bold)	Took part in previous AAA research?	ASD service history
Service 1	ASD team (ID and mental health)	Large urban	Health Board Region 1 (Population 1,196,335)	Multi-disciplinary	9	Lead **Speech and Language Therapist** (SLT), Nurses & Occupational Therapist (OT), Consultant Psychiatrists, Consultant Clinical Psychologist	Yes	Specific ASD service in place for over 10 years
Service 2	Mental health team	Urban	Health Board Region 5 (Population 400,000)	Multi-disciplinary	9	**Clinical Psychologist**, Consultant Clinical Psychologist, SLT	No	New service under development
Service 3	Intellectual disability team	Large urban	Health Board Region 1	Multi-disciplinary	2	**Clinical Psychologists**	No	General ID team doing ASD diagnosis for over 5 years
Service 4	Intellectual disability team	Large urban	Health Board Region 2 (Population 652, 230)	Multi-disciplinary	10	**Specialist Nurse**, Clinical Psychologists, General Psychiatrists, OT, Community Nurses	Yes	Specific ASD service in place for 5 years
Service 5	Intellectual disability team	Urban	Health Board Region 4 (Population 358, 900)	Multi-disciplinary	8.9 across MH and LD teams	**Clinical Psychologist**, psychology assistants and trainees	Yes	New specific ASD service under development
Service 6	Mental health team	Urban	Health Board Region 4 (Population 358, 900)	Multi-disciplinary	8.9 across MH and LD teams	**Clinical Psychologist**, psychology assistants and trainees	Yes	New specific ASD service under development
Service 7	Intellectual disability team	Large urban	Health Board Region 1	Multi-disciplinary	6	**Clinical Psychologist**, Nurse, SLT, Psychiatrist, Autism Support Co-ordinator	No	General ID team doing ASD diagnosis for over 5 years
Service 8	Intellectual disability team	Urban	Health Board Region 6 (Population 320,000)	Multi-disciplinary	3	**Professional Lead Nurse**,, Clinical Psychologists	No	New specific ASD service under development
Service 9	Intellectual disability team	Urban	Health Board Region 5	Multi-disciplinary	11	**Consultant Clinical Psychologist**, Clinical Psychologist, Social Worker, SLT, Dietician, Community LD Nurse, Consultant Psychiatrist, OT, Art Therapists, Music Therapists	No	General ID team doing ASD diagnosis for over 5 years
Service 10	Intellectual disability team	Accessible rural	Health Board Region 3 (Population 110, 200)	Single profession	2	**Specialist Nurse**, Charge Nurse	No	New ASD service under development
Service 11	Intellectual disability team	Large urban	Health Board Region 1	Multi-disciplinary	2	**Consultant Clinical Psychologist**, Speech & Language Therapist	No	General ID team doing ASD diagnosis for over 5 years

Of the 11 participating services, 4 had participated in a previous phase of research and therefore already had individualised local action plans in place (Rutherford et al. 2016b). We shared the general principles that arose across all action plans with the remaining seven services. Peer group forums were attended by a minimum of two and maximum of five practitioners nominated by each service. Each service was asked to identify a lead clinician to drive changes in their service and the professional roles of the identified leads included: a Speech and Language Therapist; Nurses and Clinical Psychologists (given in bold in Table 1).

### Policy and Funding Context

In Scotland, adult ASD diagnostic services are largely provided by the National Health Service. Healthcare is free to individuals at the point of delivery and healthcare spending per capita is close to the OECD average (OECD 2015). When there is concern that an adult needs ASD assessment, referrals are made either through a GP or by a mental health practitioner. The Scottish Government has set out a ten year national autism strategy (Scottish Government 2011) which includes the aim of improving access to diagnostic assessment and has a strong focus on developing provision for adults with ASD. In 2012 there were adult diagnostic services available in 11/14 Scottish health boards Autism Achieve Alliance (2012). Of these 11 boards, all had services providing ASD diagnostic assessment for adults with intellectual disability but only 5 had dedicated ASD diagnostic service provision for those without ID. Historically diagnostic centres were available in the main cities and adults could be expected to travel out of their authority for assessment. As recognition has risen in recent years, there is an aspiration to provide more local provision. This study was commissioned to support effective service provision in new and existing services.

### The Change Program

The program was made up of two elements: (a) the Autism Achieve Alliance (AAA) pathways and documentation which were designed to facilitate the reduction in waiting times for diagnostic assessment (AAA 2014; Rutherford et al. 2016b), (b) the change program which put targeted supports into place to accelerate the change. The program took place over a 12 month period, with three main phases: committing to change and setting plans in months 0–3; driving change in months 4–9 and sustaining change in months 10–12.

#### Pathways and Documentation

A workshop for participants was used to determine what changes were currently required within their own service that they anticipated would reduce waits. During the initial workshop participants were exposed to the local action plans which were built during previous research (AAA 2014; Rutherford et al. 2016b). The participants identified the need to develop practical pathways and documentation in order to operationalise NICE guidance and the changes identified in the previous AAA research. The pathways and documentation also incorporated screening questions for identifying the presence of risk factors for ASD; guidance on reducing the high non-attendance rates; creating routes to post diagnostic support; reducing the number of contacts; reducing inappropriate referrals; increasing the quality of information before first appointment; improving the efficiency of multi-disciplinary team (MDT) working and care pathways (AAA 2014). (See Table 2).

**Table 2:** Key objectives and solutions to reduce the wait for diagnosis

Objectives	Solution
To develop efficient working and communication by	Speeding up administrative processes, for example by using report-writing proformas; collecting and reviewing information to support forward planning of the service; having a multi-disciplinary team with dedicated time for ASD assessment and diagnosis; carrying out reviews and succession planning of training needs for each service, and opportunities for continuing professional development (CPD)
To reduce non-attendance rates by	Implementing a pro-active attendance policy as part of the care pathway, relevant to the client group
To reduce inappropriate referrals by	Providing training and information for referrers, multi-agency partners, families etc
To improve effectiveness of care pathways by	Establishing clear pathways and detailing constructive use of time and tools at each stage of the ASD diagnostic process; utilising structured processes for requesting and gathering relevant contextual information prior to attendance for diagnostic assessment

Specific service examples of actions identified are detailed in results Table 3.

**Table 3:** Action plans for adult services: specific service examples

Stage of process	Issue	Services which identified each solution
At all stages	Care pathways	Use a clear pathway for the multi-disciplinary ASD assessment process, detailing the pathway from referral to sharing diagnosis. Develop new/ improve current care pathway. [service 1; 3; 4; 5; 6; 7; 8; 9; 10;11]
		Implement diagnostic pathway to inform assessment process, e.g. appropriate assessments to use in particular situations, minimum no. of required appointments and their purpose. [service 2]
		Improve referral procedures. Make the referral and diagnostic pathway and referral proformas available to referrers. Apply an open referral system. [service 1; 2 ;3 ;4; 8; 10; 11]
		Set time targets for completion of stages of diagnostic assessment process from referral to sharing diagnosis. [service 2; 3; 4; 8]
		Review admin processes/ Ensure adequate administrative support/ delegate admin tasks. [service 3; 4; 5; 6; 7; 10; 11]
		Develop/use report writing template to reduce time taken and improve consistency/ quality of reports and adherence to NICE guidelines. [service 1; 3; 4; 7; 10]
Pre-referral	Inappropriate referrals	Reduce number of inappropriate referrals. Provide information/ training/ leaflets/posters about indicators of ASD to referrers and potential referrers. Find out where to get a list of GPs/ Referrers in locality. [service 2; 3; 5; 6 ;7 ; 8; 10; 11]
		Introduce ASD screening questions for all referrals to the ID team. Screen existing clients in ID service to identify whether ASD assessment is indicated [service 3; 4; 5; 10]
	Limited information pre-referral	Improve quality of information received from referrers. (For example: ensure submission of screening tools with referrals/ Provide AQ-10 forms for all referrers; Send EDQ and AQ with first appointment letter). [service 1; 2; 3; 6; 7; 8]
		Provide basic ‘ASD awareness’ training to referrers. Broaden training team across the MDT to share the load [service 1; 2; 10; 11]
		Develop/ Use proformas for individual, family/ carers or referrers to complete and submit with referral form. [service 2;11]
		Request medical notes or other historical notes on acceptance of referrals. [service 1; 2]
Referral to 1st appointment	Non-attendance	Have a system to pre-empt non-attendances (e.g. opt in letters, phone calls, text messages etc.). Review non-attendance. [service 1; 3; 5; 6; 7; 10]
		Provide service in local area where possible, e.g. initial home visit. [service 1; 3; 4]
		Where appropriate enlist carer or support worker to facilitate attendance and/ or to come with the client to the appointment (e.g. where individual has an intellectual disability). [service 5]
	Reducing wait for 1st appointment	Have identified ASD diagnosis appointments to slot referrals into. [service 1; 4; 5; 6] Make appointments immediately on receipt of referral. [4; 7]
		Use information provided pre-referral to inform diagnostic process. Use screening tools (if not completed by referrer). [service 1;2; 3; 4; 5; 6; 7; 8; 9; 10;11]
	Constructive use of time	Use proformas (for observation, contextual assessment, and clinical history) during assessment and ensure these are available to all staff. [service 2; 5; 6; 7; 9; 11]
		Request that individual, family, referrers or others, as appropriate complete pro-forma requesting relevant developmental and contextual information, prior to 1st appointment. [service 2; 3; 4]
		Develop and implement an abbreviated pathway for those who clearly meet criteria for diagnosis/ less complex cases. [service 4]
First appointment to diagnosis shared	Promote effective multi-disciplinary working	Improve information sharing processes to improve MDT working. [service 3; 5; 6; 8; 9] Have dedicated, protected time for regular scheduled multi-disciplinary review meetings/ case discussions (increased frequency, shorter duration). [service 1; 4]
		Have a multi-disciplinary assessment. Work in conjunction with other diagnostic practitioner(s), with protected and scheduled slots to carry out assessments together. [service 1; 2; 9]
		Complete the diagnostic process in one day (if appropriate). [service 2]
Post diagnosis	Information	Review post diagnostic information provided. Develop/ start using packs for individuals/ carers. Engage with 3rd sector providers of post diagnostic services. [service 2; 3; 5; 6; 8; 9; 10; 11]
Quality	Training	Seek ADI-R training. [service 1; 4 ;7] Seek ADOS training. [service 1; 4; 5; 6; 7; 8]
		Provide autism awareness training in all local teams [service 2]
		Use British Psychological Society ASD Modules to improve knowledge in MDT [service 2]
		Use hints, tips and use resources shared at AAA contact days, Share AAA information with wider team [service 5; 6; 9]
	Local audit	Find out who provides ASD diagnosis in this locality [service 2]
		Find out whether the health board is interested in developing a diagnostic service for adults without ID [service 4]

#### The Flightgate Practice Development Intervention (Melton et al. 2010)

This model proposes four key practice development interventions which activate mechanisms for change. These were operationalised as follows:

### Practice Development Mentorship

Once a plan outlining the focus of change for each service was in place (as outlined in Tables 2, 3), staff support for implementing change was provided through an allocated mentor who maintained weekly contact by telephone to review the data sent in by sites to support problem solving. Mentors (research team members with clinical experience in NHS ASD services) made site visits to support the practical aspects of the changes. During the implementation phase the services returned data for each individual referred for ASD assessment using the data extraction tool outlined below. In this context mentorship referred to providing support around interpretation of data and solution focussed discussion around the practicalities of implementing the action plan.

### Practise in Practice

This was established during months 4–9 whereby it was expected that participating practitioners would be changing their practice. This involved: taking on new roles, such as adopting leadership roles; adopting new working routines with regard to ASD diagnosis, as described in their action plans and gaining an understanding of what was working and not working within their services.

### Peer Group Forums

This included an internet site to share materials and have virtual discussions alongside workshops with all participating practitioners. There were 3 workshops over 9 months, entitled (i) ‘Committing to Change’ which facilitated reflection on practice; building shared resources for change, such as structured pathways and documentation; and building a community of practice, (ii) ‘Driving Change’ addressed the actions that were put into place. Progress on change was shared with participating practitioners, (iii) ‘Sustaining Change’ reviewed the experiences and outcomes of the intervention and plans for maintenance of changes were made.

### Public Validation Roles

Leadership roles were established with participating practitioners who were changing their practice successfully in order to support other practitioners within and out with their service. Three workshops supported validation amongst peers. Opportunities for some to share their experience of introducing ASD service changes more publicly occurred at the end of the project (see Table 4).

**Table 4:** Flightgate program

Flightgate program	Description
Practice development mentorship	Provides the opportunity to debrief from baseline training, gain reassurance, advice and guidance and promote reflection, which builds confidence in using new working practices
Practise in practice	Supports integration of new knowledge and skills into practice through experiential learning. This may be achieved through shadowing others, trialling new techniques, taking on new roles to utilise new skills and self-directed learning and reflection
Peer group forums	Provides opportunities to debate successful ways of integrating new working practices. Taking on different roles within the forum will support development. This may be attending and facilitating forums, presenting successes or leading group activities
Public validation roles	Engaging in additional or enhanced roles related to new working practices provides intrinsic reward and enhanced motivation to continue with own development and to support others. This may include offering practice development supervision, taking on a training role or sharing expertise and best practice through publications and presentations

### Procedures

The study followed a number of steps. All services in Scotland offering regular diagnostic assessment to adults with possible ASD were eligible to participate (n = 32). These services were identified from the Diagnosing Services National List, which acted as the sampling frame. Eleven services accepted the invitation and the research team met with them initially to establish their expectations of participation. Baseline data from casenotes (n = 71) of individuals referred to these services for ASD assessment in the preceding 24 months were then collected prior to the introduction of the change programme. Follow up data were collected from each of the cases (n = 88) referred for ASD assessment to each service after implementation of the change programme for comparison.

### Data Collection

#### Casenotes

Casenotes were included for analysis if (a) the individual had been referred for an assessment of ASD, regardless of the final diagnosis (b) the individual had been referred to the service within the past 24 months. An individual case note data extraction tool was adapted and shortened from the tool used in previous research, in order to make its use feasible in clinical practice for every case included (McKenzie et al. 2015). The tool recorded the demographic details of the individuals, the time between referral and receiving diagnosis and components of diagnostic assessment. Data were gathered by the assessing practitioner on 159 case notes (71 before the change program and 88 after the change program). Pre and post individual case note extraction forms were excluded from some elements of analysis if they contained missing data (total n = 13). Whether cases came from urban or rural areas was calculated based on postal code (Scottish Government Urban Rural Classification 2012–2013). There were 36% of cases from large urban areas; 46% from other urban area; 18% from accessible rural areas.

During implementation, data extraction forms were returned by services on completion of each assessment. Within 1–2 weeks of receipt, these were analysed and used to provide feedback to services to support solution focussed ongoing, timely monitoring and implementation of action plans.

#### Services

Services also completed a service data extraction tool to provide data about service configuration, the diagnostic process and pathways in each service. Service configuration details are summarised in Table 1.

### Data Analysis

Our data involved a nested structure with cases nested within services. To account for this nested structure we analysed our data using a series of model comparisons within multi-level model framework. Of primary interest was the question of whether the change program had successfully reduced wait times for ASD diagnosis. Our level-1 units were individual cases, our level-2 units were services, our predictor was time point (pre- versus post-change program) and our outcome was wait time. All models were estimated using maximum likelihood estimation using the ‘nlme’ package in R statistical software (Pinheiro et al. 2013).

We first evaluated whether there was a substantial effect of clustering on our data by fitting a baseline intercept only model in which a fixed effect for the intercept only was estimated. This model, which includes no random effects or predictors, is also sometimes known as an ‘empty model’. We compared the fit of the intercept only model to a random intercepts model in which a random effect for the intercept is estimated. If the latter is better fitting, this implies that the effect of clustering is non-trivial and should be taken into account. Here we judged ‘better fitting’ to be a model in which parsimony corrected fit indexes Akaike’s Information Criteria (AIC; Akaike 1987) and Bayesian Information Criterion (BIC; Rafferty 1995) were smaller than the comparison model. Therefore, if the addition of random intercepts resulted in a better fitting model, then we proceeded to test our substantive hypothesis regarding the effect of the change program on wait times by adding our predictor (time point) to the random intercepts model to give us a random intercepts fixed slope model.

## Results

### Waiting Times

Table 5 illustrates some of the characteristics of the sample. There were more males with ASD than females. The pre-change program sample comprised 39 males and 25 females (data were missing for 7). The post- change program sample comprised 62 males, 24 females and 1 transgender individual (data were missing for 1). The average referral age for the pre-change program sample was 31.3 years (SD = 11.7) and for the post- change program group 30.2 years (SD = 10.5). In the pre-change program sample, the mean age of diagnosis of ASD was 31.5 years (SD = 11.6) and 31.0 years (SD = 10.7) for the post-change program sample. There were no significant differences in age of referral or diagnosis between the pre and post—change program samples. Individuals were considered to be at increased risk for ASD, due to presence of factors such as having a learning disability, additional support need or family history of ASD, in 77.5% (55/71) of pre-change program cases and 52.3% (44/84) of the post-change program cases. There were 36% of cases from large urban areas; 46% from other urban area; 18% from accessible rural areas.

**Table 5:** Pre and post change samples

	Pre (total n = 71 cases)	Post (total n = 88)
Male	39	62
Female	25	24
Transgender	0	1
Missing	7	1
Gender ratio	1.6–1	2.6–1
Mean age at referral (SD)	31.3 years (70/71, missing data) 11.7	30.2 years (88/88) 10.5
Mean age at diagnosis (SD)	31.6 years (68/71, missing data) 11.7	30.8 years (85/88, missing data) 10.6
Increased risk of ASD	55/71	*44*/*84* (missing data)
Diagnosed with ASD	52/71	*42*/*88*
No ASD diagnosis	15/71	*43*/*88*
Assessment incomplete or inconclusive	4/71	*3*/*88*

Descriptive statistics by service are provided in Table 6. Wait times were non-normally distributed (skew = 2.32, kurtosis = 9.80), therefore, we used a natural log transformation of this variable for subsequent analyses. This successfully dealt with the non-normality.

**Table 6:** Descriptive statistics by service

Service	Pre-flightgate program		Post-flightgate program	
	N	Mean (SD)	N	Mean (SD)
1	17	146.5 (75.8)	51	113.9 (55.9)
2	8	122.3 (41.8)	7	79.4 (44.8)
4	10	128.8 (47.2)	11	103.4 (37.4)
5	2	430.0 (260.2)	2	68.5 (0.71)
6	5	190.6 (73.7)	6	243.8 (97.6)
8	8	121.1 (42.4)	4	93.0 (65.3)
9	3	115.7 (16.8)	1	146 (N/A)
10	5	189.4 (34.4)	1	229 (N/A)
11	4	107.5 (87.8)	1	190 (N/A)
Overall	62*	149.4 (86.4)	84*	119.5 (67.4)

^a^Due to missing data from the pre and post Flightgate Programme samples, the analysis to measure the reduction in wait times is based on:

62 pre intervention cases and

84 post intervention cases

Due to the variability in number of referrals between services, it is was not possible to undertake further statistical analysis to understand individual factors which affected whether waiting times increased or reduced within individual services. Of the 11 participating services, 2 ID services had no referrals during the post data collection phase, 5 services reduced their wait time (1 ID and MH team; 1MH and 3 ID) and 4 increased their wait time (3 ID and 1 MH). The 3 services with the highest total number of cases referred (services 1, 2, 4) all reduced their mean waits. They provided data for 71% (n = 104/146) cases. None of the 3 services with the lowest number of total cases referred (where they had both pre and post data) reduced their waits.

### Multi-level Models

The random intercepts model was better fitting than intercept only model (Δχ^2^ = 7.40, p = 0.007, ΔAIC = 5.40, ΔBIC = 2.42; positive changes indicate a better fitting model), suggesting some effect of clustering was present. The ICC was 0.17. The random intercepts fixed slope model fit better than the random intercepts model (Δχ^2^ = 8.15, p = 0.004, ΔAIC = 6.15, ΔBIC = 3.16), suggesting that the intervention variable explained variability in wait times. The effect of intervention on wait times in this model was statistically significant (b = − 0.25, t(136) = − 2.88, p = 0.005). This suggests that the change program significantly decreased wait times. As a robustness check, we also estimated a random intercepts fixed slope model using the raw (untransformed) wait times. The effect of the change program was also statistically significant in this model (b = − 28.31, t(136), p = 0.02) (see Table 7).

**Table 7:** Model fits for multi-level models

Model comparison	ΔAIC	ΔBIC	Δχ^2^	p
Intercepts only versus random intercepts	5.40	2.42	7.40	p = 0.007
Random intercepts versus random intercepts with fixed effect for time	6.15	3.16	8.15	p = 0.004

Positive changes indicate a better fitting model

The longest service wait was reduced by 52 weeks (12 months). The average amount of time an individual waited for diagnosis across all services prior to the change program was 149.4 days (21.3 weeks). The average wait after the change program was 119.5 days (17 weeks). Therefore, there was an average reduction of 29.9 days (4.3 weeks) in overall waiting times between pre and post the change program for the period between referral and sharing the diagnosis.

### Process and Pathways, Mentorship and Practise in Practice

During implementation services returned case by case data extraction forms each week. The research team then analysed this data for discussion and shared written and verbal feedback. Services were told the results, i.e. the duration of each part of the assessment (T1—from referral to 1st appointment; T2—1st appointment to diagnosis; T3—diagnosis made to diagnosis shared and T4—Total duration). The current week’s results were compared with targets set and duration for this service prior to implementation. This was used to maintain focus and motivation on targets and agreed actions and how these were working.

### Service Configuration and Team Working

In this study, there was variability in the number and range of professionals in each team across sites and we were unable to identify whether there was any relationship between team make-up or the professional leading each assessment and length of process. The core assessment was usually led by one clinician, who complete the clinical history information gathering; contextual assessment (via observation, questionnaire or interview) and direct observation in the clinical setting. Additional assessments from other team members, where appropriate contributed relevant information or added to the length of the assessment period (e.g. cognitive assessment, communication assessment, mental health assessment). Some teams did clinics jointly, in pairs. Most services reviewed assessment information at team diagnostic formulation meetings and then the key clinician or caseworker fed back the diagnosis reached.

## Discussion

There is an identified need to provide improved diagnostic assessment for adults with autism. Although further research is needed, this study goes some way to providing evidence for service providers about which factors might support positive change through applying implementation science to the ASD context.

The study aimed to examine the effect of the Flightgate practice development interventions (Melton et al. 2010) combined with ASD specific evidence based pathways and documentation (Rutherford et al. 2016b), in reducing the wait time between referral and sharing the outcome of ASD diagnostic assessment in adults. Given that the findings suggest an average reduction of 29.9 days (4.3 weeks) in overall waiting times was possible, this approach may be a way forward to facilitating quicker access to diagnosis and support for adults with ASD. It should be noted that one service, with a small number of referrals each year reduced its wait by 52 weeks, which represents a substantial reduction in wait for service users accessing this service.

Delayed diagnosis and the effects of this can extend well into adulthood for many people with ASD (Taylor and Marrable 2011; Brugha et al. 2011). This study demonstrated that the services completing assessment for adult diagnosis of ASD had an average wait time of 149.4 days and, following a targeted change program, were able to reduce this to an average wait time of 119.5 days. This latter figure is in line with the recommended maximum wait time for ASD diagnostic assessment in children of 119 days from referral to diagnosis (Le Couteur 2003). Without a control group we cannot confirm fully that the change program was the cause of this change and further research would be of value, to explore the effects of this change model applied in other ASD diagnostic services with adults and children.

Diagnosing ASD as early as possible is beneficial to individuals and their families, because it helps explain the challenges they face and it improves access to relevant and effective supports. Increasing awareness of ASD in adults has the potential to increase referral rates considerably (Gallagher et al. 2013) and therefore services may seek to utilise the current research findings to review efficiency of ASD diagnostic pathways, together with consideration of applying clinical guidelines.

The changes targeted within this study supported the adoption of evidence based practice (Drake et al. 2001) and were expressed in local actions plans. Specific local targets were identified through review of service data alongside NICE 142 guidance (2012) and the aggregated local action plan from the Autism Achieve Alliance (2014) and Rutherford et al. (2016b). While clinical guidelines can support clinicians in providing a high quality and consistent standard of care, there are recognised challenges to their successful implementation (Ltd 2005).

There is strong evidence from practice that neither motivation nor knowledge that change is needed are sufficient to deliver sustained and effective change (Melton et al. 2010) and that an organised process or framework is required for implementation (Meyers et al. 2012). It was necessary to look beyond the published evidence within the ASD literature to address the problem of delayed diagnosis in adults with ASD and to incorporate evidence from implementation science literature to identify a framework for practice development and service change. The Flightgate intervention (Melton et al. 2010) potentially provides an evidence based model to support successful implementation of action plans into everyday practice by providing important practical tools to operationalise the NICE guidance.

The elements used within the change programme in ASD services were consistent with the multi dimensional Flightgate intervention (Melton et al. 2010) suggesting that it may have utility as a framework for promoting changes in clinical practice in NHS diagnostic services. Research practice partnerships support implementation of evidence based practice and confer reciprocal benefit of collaboration (Aarons et al. 2011) through incorporating practice development mentorship, practise in practice, peer group forums and public validation as core tenets of the Flightgate framework (see Table 4).

In common with previous studies of actively delivered, tailored and targeted change programs (Dobbins et al. 2009), the responsive model used in this study integrated literature evidence with several strategies and participants’ feedback in co-creating locally relevant decisions about ‘what to change’ (Blank et al. 2014; Park et al. 2014). To support services with ‘how to change’, research practitioners (with experience of working within ASD services and knowledge of the change program) provided practice development mentorship. This type of supportive feedback mechanism is common in implementation studies (Meyers et al. 2012). The present study included regular opportunities to reflect and to gain reassurance and advice, together with support to successfully change practice, take on new roles and adopt new routines and knowledge (Melton et al. 2012). Clinical networks make communication infrastructures more readily available than workshops and educational sessions alone and allow for both research evidence and expertise about its clinical application to be effectively shared amongst practitioners (Conklin and Stolee 2008; Forsetlund et al. 2009). In the current study, peer group forums promoted learning through interacting with others. This was both face to face in workshops and electronically using a shared internet space. Public validation is important for maintenance and sustainability of change (Melton et al. 2012) and future evaluation may provide evidence on how the services sustained the changes without regular contact with the research team.

In this study the variability in service configuration and in referral rates between services, limits our ability to use statistical techniques to understand which individual service and intervention factors specifically had the greatest impact. We can hypothesise that from the data available that services with higher referral rates would have benefited more from the opportunity afforded by the Flightgate model: (1) they had more frequent practice development mentoring and case by case discussions about how well changes were working, opportunities for reflection and the chance to implement the action plans made. (2) Integration of new knowledge requires practise in practice and therefore if a service has few or no referrals, they are limited in taking up this aspect of the framework. (3) Staff are more likely to engage in peer group forums if they have current and relevant queries or ideas to share. (4) There is a potential challenge in public validation and motivation to sustain changes when in a service with low demand for this specialist skill. In future, services wishing to undertake a similar process may wish to ensure they have sufficient referrals to allow clinicians to benefit from the model.

Interestingly the services with the lowest referral rates were 5 ID services. One participant reflected that in recent years they had done a lot of ASD diagnostic assessment but perhaps have now “caught up” with older adults with ID who had been missed. Now, younger adults transitioning from child services with ID mostly have their ASD identified. This is an interesting proposition and may be worth further enquiry.

One service reduced the mean wait time by 52 weeks, with only a small number of cases referred (i.e. 2 referrals each in the pre and post phase). Table 5 highlights that this service was a new LD service. Once again, we can only hypothesise but there is a possibility that such large reductions may be more likely when there has previously been no service and a new pathway is implemented. Longstanding services, with larger referral rates may expect to see smaller mean difference, more like those in service 1 (reduced from 146.5 to 113.9) and service 4 (reduced from 128.8 to 103.4).

Services for adults with ASD have been under-researched to date (Howlin and Moss 2012; NICE 2012). To our knowledge this is the first study to report on the implementation of diagnostic service improvement for this population. The conceptual framework applied, which led to successful reduction of the wait for ASD diagnostic assessment, makes a novel and an important addition to the literature in this field. The study successfully embedded the implementation model within quantitative data collection and analysis procedures.

### Implications for Practice

Variations in how ASD services are funded within and between nations can create complex paths to diagnostic services (Iacono et al. 2017) however, the challenge of meeting the needs of adults who require access to ASD diagnostic assessment is present regardless of the healthcare system in place.

The intervention has clinical relevance for both generalist and specialist adult or child services and for those focused on the assessment for possible ASD in individuals with or without an intellectual disability. It could be replicated and further evaluated in other child or adult services interested in an evidence based model to reduce the wait for ASD diagnosis. It acknowledges that one size does not fit all and that each service will have different priorities for change, whilst signposting to improvements other services have found helpful. It uniquely combines expertise in ASD with expertise in the science of effective service change, to offer a practical option for clinical services. Combining this intervention with adherence to clinical guidelines would support delivery of a high standard of care (McKenzie et al. 2016). Given the well documented challenge of integrating research evidence and clinical guidelines into practice (LaRocca et al. 2012), the results of the present study, following on from the publication of the NICE (2012) and SIGN (2016) adult ASD guidelines, suggest that this model, with its strong theoretical framework (Park et al. 2014) has the potential to support the implementation of evidence based practice in other ASD services and healthcare settings.

### Limitations

The primary limitation of the current study was the lack of a control group to ensure that any changes observed over time were due to the intervention. For example, it is possible that at least part of the effect of the intervention was due to the fact that the services knew their performance was being monitored by virtue of their participation in the study. Related to this is the possible self-selection of participating services i.e. that those services who agreed to participate in our intervention were the most motivated to change, thus potentially leading to an overestimate of its effectiveness by excluding services that were less likely to engage with and benefit from the intervention. Future studies would benefit from using a randomised controlled trial design to determine whether the benefits found in this study are repeated under more controlled conditions.

## Conclusions

This study provides evidence that applying the change program following the Flightgate change process together with changes within pathways and processes was associated with reduced wait times for ASD diagnosis.
